# A Charging-Aware Multi-Mode Routing Protocol for Data Collection in Wireless Rechargeable Sensor Networks

**DOI:** 10.3390/s19153338

**Published:** 2019-07-30

**Authors:** Shih-Chang Huang

**Affiliations:** Department of Computer Science and Information Engineering, National Formosa University, Yunlin 632, Taiwan; schuang@nfu.edu.tw

**Keywords:** charging-aware multi-mode, data gathering, multi-level convex hull circle, life-delay ratio, wireless rechargeable sensor networks

## Abstract

This paper proposes a charging-aware multi-mode routing protocol (CMRP) to collect data in the wireless rechargeable sensor networks. The routing mechanism in CMRP is not steady but changes according to the energy charging status of sensors. Sensors that cannot replenish their energy efficiency use the routing protocol with less energy consumption. On the contrary, sensors that can replenish their energy use the low propagation delay routing protocol. A novel heuristic chaining mechanism based on multi-level convex hull circle (MCC) is also proposed. Simulation results show that CMRP not only has longer operation time than LEACH and PEGASIS but also has the shortest propagation delay time. The lifetime of CMRP is less than the minimum spanning tree by about 1%, but the propagation delay is shorter than MSTP about 21–28%. In addition, CMRP considers both reducing energy consumption and shortening the propagation delay at the same time. The life-delay rate of the CMRP is close to the optimal results.

## 1. Introduction

Wireless sensor networks are popularly applied for variant environmental monitoring applications such as forest fire detection [1], intruder detection [2], and factory or pasturage monitoring [3]. Sensors are tiny devices that consist of multiple sensing components, a simple computation unit, and the wireless communication component [4]. The energy source of the sensors usually comes from their installed batteries. Using the limited energy resource efficiently is a critical factor to prolong the operation time of the sensors.

Many energy-aware data gathering routing protocols have been proposed to prolong the operation time of the wireless sensor network. These studies can be classified into two classes. The first class is cluster-based routing protocols [5,6,7,8,9,10]. Sensors in this class are organized into multiple clusters, and each cluster selects a cluster head (CH) as the coordinator to handle the communication sequence and to aggregate the data of the members. After each CH aggregated the data of all cluster members, the collected data is transferred to the sink which is a data center of the network.

The second class is chain-based routing protocols that sensors are organized in the chain format [11,12]. Sensors are chained as a Hamilton path. One of the sensors is selected as the leader, which is similar to the CH in the clustering methods to aggregate the sensors’ data and to relay it to the sink. The data of sensors is propagated to leader hop by hop according to the sequence of the chained format. The number of sensors relaying data to sink reduces to one in the chain-based routing protocols. Similarly, the leader in the network is also periodically changed to share the relaying overhead.

In addition to the above two class, some studies use movable vehicles to be the mobile sink to visit the sensors and gather their data [13,14,15,16,17]. Furthermore, some studies focus on visiting and charging the sensors [18,19,20] that is planning the routing path of the chargers. They can also be applied for data gathering when they charge the sensors. These kinds of methods generate much propagation delay time. They are not suitable for applying in large-scale networks and for the sparse deployed sensor networks. Although many mobile charging methods have already proposed to replenish the energy of sensors, they are not applied for the scenario that sensors are stationary collecting data continuously.

The above methods are intuitively designed for the sensors that cannot replenish energy. Directly applying these methods to the rechargeable wireless sensor networks may lower the utilization of energy.

Therefore, this paper proposes a charging-aware multi-mode routing protocol (CMRP) to collect data in wireless rechargeable sensor networks. The proposed CMRP method dynamically changes the data propagation mechanism according to the charging status of the deployed sensors. CMRP makes the sensors which are not in the charging area save their energy and lets the sensors which are in the charging area quickly propagate data to sink. It considers both the network lifetime and propagation delay.

The rest of this paper is organized as follows. The related studies are reviewed in Section 2. The proposed charging-aware multi-Mode data routing protocol is provided in Section 3. Section 4 presents the simulation results to compare CMRP with the existing methods. Finally, the conclusions are presented in Section 5.

## 2. Related Works

Many data collecting routing methods have been proposed. These methods can be classified into two categories, cluster-based and chain-based. In cluster-based methods [5,6,7,8,9,10], the sensors are divided into several clusters. Each cluster elects a cluster header (CH) to serve as the local base station. Other sensors in this cluster forward their data to the CH directly. Next, each CH forwards the aggregated data to the sink. To balance the energy overhead of being the CH, each cluster elects a new sensor to be the cluster head for service. There are many clustering mechanisms proposed for data gathering. Sensors may be organized into constant size clusters [5], clustered according to the grids that they are deployed [6], based on the fuzzy logic or fuzzy c-mean mechanism [7,21], or type-2 fuzzy logic and ACO [22]. In addition, clustering sensors according to the sensors’ energy in heterogeneous wireless sensor network is also proposed [23]. The first proposed and well-known method is the LEACH approach [9]. The clustering-based methods use multiple hops propagation to shorten the transmitting distance from each sensor to the sink directly. It can also prevent all sensors from consuming a lot of energy on long-distance data transmission except the sensors being the CHs. Therefore, the sensors with plenty of energy are the candidates to be the cluster head [14].

The cluster-based methods can be improved further if the number of CHs can be reduced. All sensors can aggregate their data to one sensor and then transmits to sink. Sensors can be organized into the chain format to reduce the data propagation distance to the next hop. In the chain-based methods [11,12], each sensor only has one or two neighbors. Sensors that located at both ends of the chain have one neighbor and the others have two neighbors. Each sensor combines its data with one neighbor’s data and then forwards to the other neighbor. Eventually, a sensor named ‘leader’ aggregates all data and transferred to the sink. Similar as the cluster-based methods, sensors take turns to serve as the leader to share the long-distance transmission overhead. The well-known chain-based method is PEGASIS [11]. 

The chain-based protocols do efficiently reduce the energy consumption of sensors spent on long-distance data propagation, but the cost is the increasing propagation hops. The number of data propagation hops increases as the number of sensors grows. In a network with *N* sensors, the maximum number of propagation hops is *N/2* when the leader is located at the middle position of the chain. However, the maximum number of propagation hops is N-1 when the sensor located at the end of the chain is selected as the leader. A variation chaining mechanism named ring-based information collection architecture (RICA) is organizing the sensors as a circle [24]. Each sensor in the circle of the RICA method has two directions to propagate its data to leader and the maximum number of propagation hops can always be *N/2* if the sensors are evenly divided between the left side and the right side of the leader.

The key issue of the chain-based methods is the chaining mechanism. Building the Hamilton path or the Hamilton cycle has already known to be NP-complete problem. Heuristic chaining mechanisms are applied to link the deployed sensors [25,26]. A worse chaining sequence may cause the sensors to exhaust their energy faster than the sensors in the cluster-based methods. Another variation of the chain-based methods is the tree-type methods [27,28,29,30]. The tree-type methods use the minimum spanning trees (MSTP) [17] to create the shortest link between the sensors and their neighbors or try to propagate data to sink with a shortest distance path. The problem is the maximum number of propagation hops of the minimum spanning trees is topology dependent, and greedily using the shortest path causes the sensors exhaust their energy quickly.

Although some of the studies use movable vehicles to be the mobile sink to visit the sensors and gather their data [13,14,15,16,17]. These kinds of methods generate much propagation delay time when sensors wait the mobile chargers to move to neighborhood. They are not suitable for applying in large-scale networks and for sparsely deployed sensor networks.

## 3. Charging-Aware Multi-Mode Routing Protocol

### 3.1. Preliminaries and Assumptions

The proposed method has the following assumptions. Sensors are deployed within the region of interest (RoI) randomly and are stationary after they are deployed. Their locations are reported to the sink. The sink will announce the network topology information to all sensors in the network. The coverage of sunlight only includes parts of the RoI and regularly transfers to another part after a period of time. The coverage of sunlight only transfers to a nearby area. The communication ranges of the sensors are uniform. Every sensor installs a solar panel to replenish its energy when it is under the sunlight coverage. Each sensor has a unique identity, and the location of the sink is at the center of the RoI.

According to the energy replenish statue of sensors, they are classified into three types. The sensors covered by the sunlight are classified as the type1 sensors that they can replenish their energy. By having plenty of energy resources, these sensors can use the routing protocols which are more generous on energy consumption to reduce the data propagation delay. The cluster-based routing protocols have the fewest propagation delay can be applied for these sensors. The sensors in the area attached to the sunlight coverage are type2 sensors. These sensors cannot replenish their energy at this time. They are the sensors which the sunlight coverage just left them or will be covered by the sunlight. These sensors own good locations to communicate with the type1 sensors. The ring-based routing protocols that consider both energy usage and the propagation delay simultaneously are the candidates.

The type3 sensors are those neither the type1 nor the type2 sensors. These sensors will endure a long time before the next time the sunlight coverage includes them. Their energy needs to be used well to prevent from exhausting their energy. The routing protocols with the best energy efficiency are the best solution. The spanning tree routing protocols are the candidates.

Sensors periodically return their gathered data to sink. A round is defined as the sink receives the collected data of all sensors. In one round, the data propagation sequence is according to the type of sensors. The type3 sensors are the first group to propagate data; the next is the type2 sensors and so on. The next round starts when the sink receives all data of sensors.

#### Radio Energy Model

The radio energy model of sensors used in this article is the same as [5]. Each sensor can adjust its transmission power to the necessary energy level while sending data to its recipients. Let *E_elec_* = 50 nJ/bit be the energy consumption to switch on the transmitter (or receiver) circuits to dissipate one bit of data. The energy consumption for transmitter amplifier to dissipate one bit of data is denoted as *ε_amp_* = 100 pJ/bit/m^2^. The radio loss is proportional to the square of the distance under the assumption of a free-space environment. Let *E_tx_* (k, d) be the energy that a sensor transmits a k-bit message to a receiver with distance d. Each sensor needs to switch on the transmitter circuits and the transmitter amplifier when it sends data. *E_rx_* (k) is the energy that a sensor receives a k-bit message. Each sensor needs to switch on the receiver circuits for receiving data. They are computed as (1) and (2).

*E_tx_* (k, d) = *E_tx-elec_* (k) + *E_tx-amp_* (k, d) (1)

*E_rx_* (k) = *E_rx-elec_* (k) (2)

where *E_tx-elec_* (k) = *E_elec_* * k, *E_tx-amp_* (k, d) = *ε_amp_* * k * *d*^2^ and *E_rx_* (k) = *E_elec_* * k.

Each sensor can aggregate the received data from several sensors. The methods such as LEACH, PEGASIS, and RICA are focus on the energy consumption by reducing the average transmission distance. The CMRP approach proposed in this paper also attempts to reduce the average transmission distance and propagation delay

### 3.2. Charging-Aware Multi-Mode Routing Protocol (CMRP)

Each sensor maintains several different propagation targets for different charging status. When a sensor is not within the sunlight coverage, it needs to save as more energy as possible. The minimum-transmitting spanning tree (MSTP) with sink as the root is the best solution. This method can reduce the propagation distance to save energy. [27]. All sensors are included to construct the minimum spanning tree (MST). Each sensor *S* stores its parent node, denoted as *P*(*S*), according to the established MST. The purpose that includes all sensors to construct the MST instead of only the type3 ones is to shorten the distance between the linked sensors. Because the sunlight coverage transfers periodically, involving all sensors to build the MST can help sensors reduce the propagation distance to the adjacent type of sensors. Thus, the next hop that sensor *S* passes its data to sink, denoted as S^*^, is represented as (3).

(3) $$S^{*}=P\left(S\right),cls\left(S\right)\in type-3$$

Next, all type1 and type2 sensors are included to build the circular chain. Involving two types of sensors can reduce the average data propagation distance of the type2 sensors. Furthermore, the type1 sensors which have plenty of energy can be selected the leader to prolong the operation time. Let *C =* {*v*_1_, *v*_2_, *v*_3_, *…*, *v_n_*} be the built circular chain with *n* sensors in it. The leader, denoted as *v^L^*, is selected based on the Equation (4).

(4) $$v^{L}\;=Max_{i}^{n}\left(E\left(v_{i}\right)+Ec\left(v_{i}\right)\right)|v_{i}\in C$$

where *E*(*v_i_*) is the residual energy of sensor *v_i_* and *E_c_*(*v_i_*) is the amount of replenished energy that sensor *v_i_* charges from current time to the end of the charging period.

Furthermore, instead of using the regular hop by hop propagation on the circular chain, the CMRP does some modifications to reduce the propagation delay. The sensor *v_i_* is the current leader of the circular chain *C*. Sensor *v_j_* requires finding its next hop, denoted as sensor v_j_^*^, to deliver its data to sink. Let *m*_∆_ = $\lceil \frac{n}{2}\rceil -i$. *m*_∆_ is the offset to adjust the leader to the middle position on the chain *C*. The adjust position of the sensor *v_i_* will be *v_im_*, where *im* = *i* + *m*_∆_ and the adjust position of the sensor *v_j_* will be *v_jm_* where *jm* = (*j* + *m*_∆_ + *n*) *mod n*. For the given sensor *v_j_*, its v_j_^*^, is computed as (5) to half the number of data propagation hops in the chain-based protocols [24].

(5) $$v_{j}^{*}=S_{k}^{*},\;where\;k=\{\begin{array}{c} \begin{array}{cc} \left(j+n+1\right)mod\;n, & j_{m}<\lceil \frac{k}{2}\rceil \end{array}\\ \begin{array}{cc} \left(j+n-1\right)mod\;n, & j_{m}>\lceil \frac{k}{2}\rceil \end{array} \end{array}$$

However, in this paper, sensors are rechargeable. This circular chain involves the type1 and type2 sensors. It is possible that a type1 sensor maps to a type2 receiver. For preventing the type2 sensor from consuming its energy, the type1 sensor will stop forwarding the data to its type2 receiver but storing in its local buffer. A special case is that the distance from sensor *v_j_* to the sink *S_s_* is closer than that to the sensor v_j_^*^. The sensor *v_j_*, in this case, will directly send its data to sink. The corresponding v_j_^*^ is represented as (6).

(6) $$v_{j}^{*}=\{\begin{array}{c} S_{\Delta }\;|\;\underset{S_{\Delta }\in \left\{S_{k}^{*},S_{s}\right\}}{\min}\left(\left|v_{j}S_{\Delta }\right|\right)\;,\;if\;cls\left(v_{j}\right)<cls\left(v_{j+1}\right)\;\\ S_{k}^{*}\;,\;if\;cls\left(v_{j}\right)=cls\left(v_{j+1}\right)\\ \;null\;,\;if\;cls\left(v_{j}\right)>cls\left(v_{j+1}\right) \end{array}$$

where *cls(v_j_)* represents the charging type of sensor *v_j_*.

At last, the sensors with a NULL v_j_^*^ use the clustering-based method to pass the data to the sink. These sensors with minimum depth in the MST are selected as the cluster heads, all sensors with NULL v_j_^*^ select their closest cluster head to be the propagation target. After then, the cluster heads send their data to the sink directly. The sunlight coverage transfers periodically. All sensors will reevaluate their current locations with the sunlight area when the location of sunlight coverage changes. At this time, each sensor can know which type it should be. In practical implementation, under-charging sensors can use message flooding to indicate the other sensors or the energy replenish rate can be applied to identify the corresponding type of each sensor. The algorithm is given in Algorithm 1.

**Algorithm 1.** The CMRP Algorithm.n: the number of deployed sensorsT: the time period that sunlight area changesTr: the remaining time period that sunlight area will changeS_s_: the sink nodeP(S): parent node of S according to the minimum-transmitting spanning treecls(S): Type of sensor Sp(S): the next propagation target of sensor SE(S): the remaining energy of the sensor SC(S, t): the amount of charging energy of sensor S after time period tDeep(S): the depth of S on the minimum spanning tree

Initialization
1. Build the minimum-transmitting spanning tree with root in sink.2. For each S,3.  stores the P(S),4.  set cls(S) according to the current location of sunlight

Periodically update, triggered as current location of sunlight changes
1. For each S,2.  Update cls(S) according to the current location of sunlight3. Z = {Sm}| for all sensors Sm with cls(Sm) = {1, 2}4. Link the sensors in Z as a circle by using the ring-based protocol.5. Set the next hop of each sensor in Z according to the Equation (5)

Routing
1. Elect a sensor S* in Z to be Leader where cls(S*) = 1 and max(E(S*) + C(S*, Tr))2. Set the p(S_z_) |S_z_ in Z, according to the Equation (5)3. For each S,4.  If cls(S) = 3,5.   p(S) = P(S)6.  If cls(S) = 2,7.   If (cls(S) < cls(p(S)))8.    p(S) = ${\text{S}}_{\Delta }\;|\;\underset{{\text{S}}_{\Delta }\in \left\{\text{p}\left(\text{S}\right),S_{s}\right\}}{\min}\left(\left|S{\text{S}}_{\Delta }\right|\right)$9.   Else If (cls(S) = cls(p(S)))10.     p(S) = P(S)11.    Else12.     p(S) = NULL13.   If cls(S) = 1,14.    Elect CHs S^H^ from all sensors S_i_ where cls(S_i_) = 1 && min(Deep(S_i_)) && p(S) = NULL15.    p(S) = min(|SS^H^|)

#### Chaining the Sensors in Circular with Multi-Level Convex Hull 

**Figure 1:**
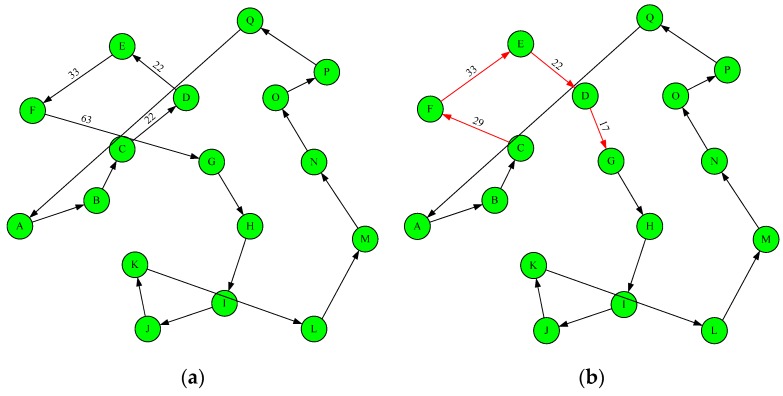

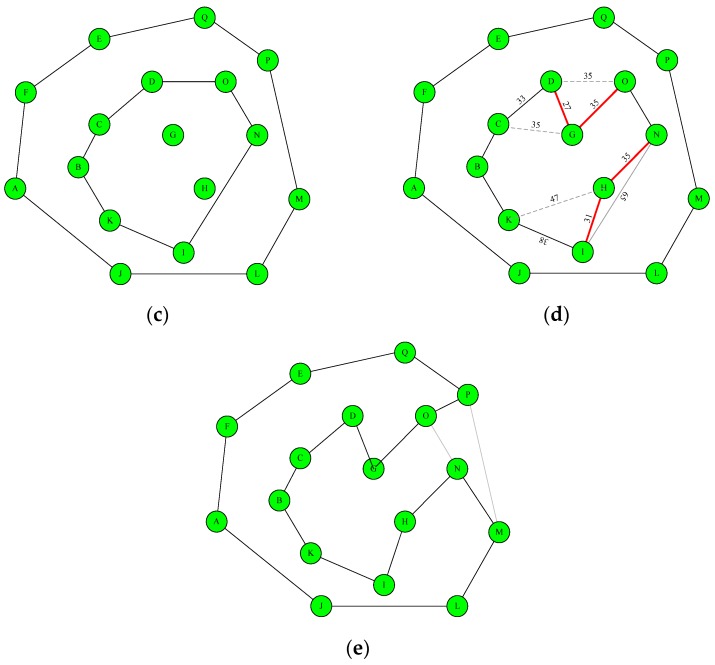
Construct regular itinerary for mobile chargers. (**a**) chain the sensors starting from node A and greedily select the next one which has the shortest distance; (**b**) remove the cross edges CD and FG in (a) to shorten the total distance of chain; (**c**) construct multiple non-overlapped circles by using convex-hull algorithm; (**d**) merge the inner nodes G and H to adjacent outer convex-hull chain; (**e**) the final merged chain of the multiple non-overlapped circles in (c).

The coordinates of sensors are the positions of the vertices on the Euclidean plane shown as Figure 1a. Chaining the vertices as a circle with minimal distance is known to be an NP-Complete problem. The heuristic method that randomly starts from a vertex and greedily selects the next one which has the shortest distance often creates a circle which at least two edges cross together like the CD and FG in Figure 1a. These cross edges indicate that at least one better solution exists to shorten the linking distance of the circle when the cross edges are removed. 

**Figure 2:**
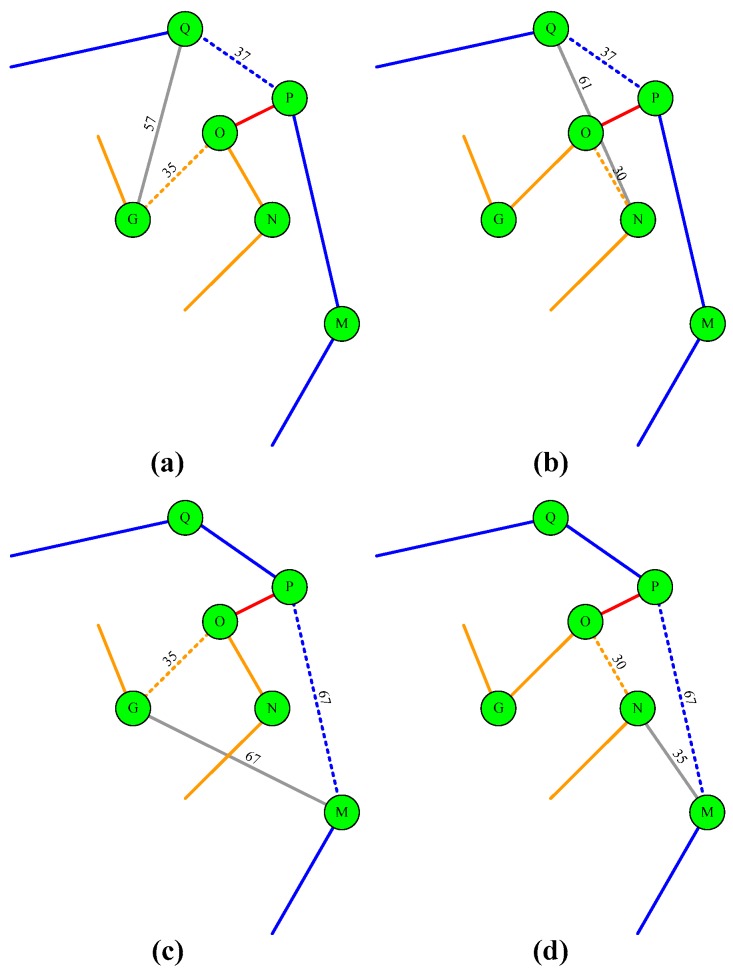
Four cases to merge two adjacent circles. (**a**) the breaking edge of the inner circle is *S_y_S_y+_*_1_ and the edge of outer circle is *S_x_S_x+_*_1_. They are $\bar{\mathrm{QP}}$ and $\bar{\mathrm{OG}}$; (**b**) the breaking edge of the inner circle is *S_y_S_y+_*_1_ and the edge of outer circle is *S_x_S_x−_*_1_. They are $\bar{\mathrm{QP}}$ and $\bar{\mathrm{ON}}$; (**c**) the breaking edge of the inner circle is *S_y_S_y−_*_1_ and the edge of outer circle is *S_x_S_x+_*_1_. They are PM and $\bar{\mathrm{OG}}$; (**d**) the breaking edge of the inner circle is *S_y_S_y−_*_1_ and the edge of outer circle is *S_x_S_x−_*_1_. They are $\bar{\mathrm{PM}}$ and $\bar{\mathrm{ON}}$.

For example, the distance of the link C-D-E-F-G in Figure 2a is 22 + 22 + 33 + 63 = 140. However, if the link changes to C-F-E-D-G shown as Figure 2b, the cross edges CD and FG can be removed. The distance changes to 29 + 33 + 22 + 17 = 101. A cross edge removing method proposed in [24] requires great computation overhead as the number of vertices increases.

The cross-edge problem occurs when the locations of the current vertex and next linked target are at the opposite side of an existing edge. This paper proposes a new vertices connection method by creating multiple non-overlapped circles from outer to inner similar to the concentric circles named multi-level convex hull circle (MCC). Then all these circles are linked into an irregular big cycle.

Let *Ψ* be the set of vertices on the plane. The convex-hull algorithm is applied to find the smallest set of vertices *Ω*_1_|*Ω*_1_ ⊂ *Ψ* which can enclose all vertices in *Ψ.* The set *Ω*_1_ in the example of Figure 1 organized the circle A-F-E-Q-P-M-L-J shown as Figure 1c. Next, the vertices in *Ω*_1_ are removed from *Ψ* and *Ψ = Ψ* − *Ω*_1_ and the vertices set *Ω*_2_ which encloses the remaining vertices in *Ψ* are computed in the same procedure. The vertices organized the circle B-C-D-O-N-I-K are included in set *Ω*_2_ shown as the inner circle in Figure 1c. This procedure continuously removes the vertices from *Ψ* until |*Ψ*| ≤ 2. Assume that the last set including the remaining vertices in *Ψ* be *Ω_k_*.

Let *Π* be the final circle containing all vertices in the network. If all vertices are removed from set *Ψ*, *Π* is initially set to *Ω_k_*. On the contrary, if the last *Ψ* is not empty, *Π* is initially set to *Ω_k_*_−1_ and all remaining vertices in *Ω_k_* are inserted into *Π*. To minimize the linked distance of the final circle, the concatenation position of each vertex in *Ω_k_* is the location that generates minimum distance. Therefore, for each vertex *S_u_* ⊂ *Ω_k_*, and each vertex *S_v_* ⊂ *Π*, the insertion point on the circle *Π* is at the vertex with minimum |*S_u_S_v_*|. The *S_u_* can be inserted in to the circle *Π* before or after the vertex *S_v_*. The decision is computed according to (7).

min(|*S_u_S_v_*| + |*S_u_S_v_*_+1_| − |*S_v_S_v_*_+1_|, |*S_u_S_v_*| + |*S_u_S_v−_*_1_| − |*S_v−_*_1_*S_v_*|) (7)

As the example in the Figure 1d, all vertices in *Ω*_2_ are initially added into set *Π.* Vertex G is one of the remaining vertices in *Ψ* and the closest vertex in set *Π* is D. The vertices before and after G are C and O respectively. The distance cost for inserting G between D and O is 27 and the cost is 29 if G is inserted between C and D. According to (7), vertex G is inserted into the circle *Π* at the position between C and O. The insertion position of vertex H is similar and the position is between I and N.

The next circle to merge with *Π* is adjacent one. The merge procedure starts from inner circles to outer ones sequentially. To merge two circles into a big one, both the circle *Π* and its adjacent circle need to break an edge. Then the two ends of their break edges are linked with each other. Let *Ω_j_* be the vertices set of the adjacent circle. Let *S_x_* ⊂ *Ω_j_* and *S_y_* ⊂ *Π.* The edge that generates minimum |*S_x_S_y_*| is the candidate one to be selected as one of the merged edge. When edge *S_x_S_y_* is selected, the other merged edge has four possible cases. They are *|S_x+_*_1_*S_y+_*_1_|, *|S_x+_*_1_*S_y−_*_1_|, *|S_x−_*_1_*S_y+_*_1_|, and *|S_x−_*_1_*S_y−_*_1_|.

The vertices {*S_x−_*_1_, *S_x_*_+1_ } ⊂ *Ω_j_*, and each of them represents the vertex in front of and in back of the vertex *S_x_* in the circle *Ω_j_*. Similarly, the {*S_y−_*_1_, *S_y_*_+1_ } ⊂ *Π*, they represent that of the *S_y_* in the circle of *Π*. The other edge is determined by the (8)

(8) $$min\left(\begin{array}{c} \left|S_{x}S_{y}|+|S_{x+1}S_{y+1}\left|-\right|S_{x}S_{x+1}\left|-\right|S_{y}S_{y+1}\right|\\ \left|S_{x}S_{y}|+|S_{x+1}S_{y-1}\left|-\right|S_{x}S_{x+1}\left|-\right|S_{y-1}S_{y}\right|\\ \left|S_{x}S_{y}|+|S_{x-1}S_{y+1}\left|-\right|S_{x-1}S_{x}\left|-\right|S_{y}S_{y+1}\right|\\ \left|S_{x}S_{y}|+|S_{x-1}S_{y-1}\left|-\right|S_{x-1}S_{x}\left|-\right|S_{y-1}S_{y}\right| \end{array}\right)$$

The procedure continues and does not stop until all circles are included into the set *Π.*

As the example in the Figure 1e, the edge $\bar{\mathrm{OP}}$, where O ⊂ *Π* and P ⊂ *Ω*_1_, with distance cost being 22 is selected as the first edge to merge these two circles. The four possible combinations of the other merge edges are given as the Figure 2a–d. In the case of 2a, these two circles are merged by adding edges $\bar{\mathrm{OP}}$ and $\bar{\mathrm{QG}}$ and removing edges $\bar{\mathrm{QP}}$ and $\bar{\mathrm{OG}}$. The distance cost is (22 + 57) − (35 + 37) = 7. The other cases in Figure 2c,d have distance costs being 16, −13, and −40. Finally, the case in Figure 2d is selected to merge these two circles because the distance cost is minimal.

The MCC is a centralized algorithm. Sink uses the positions of sensors to compute the distance between sensors and then informs each sensor its corresponding neighbors. When a sensor *S_x_* transmits its data to a dead neighbor *S_y_*, *S_x_* bypasses it and sends to the neighbor of *S_y_*. At this time, *S_x_* also marks the dead status of *S_y_* in its report data. As the sink receives this data, it will execute the chaining computation and updates to all sensors.

### 3.3. Example

**Figure 3:**
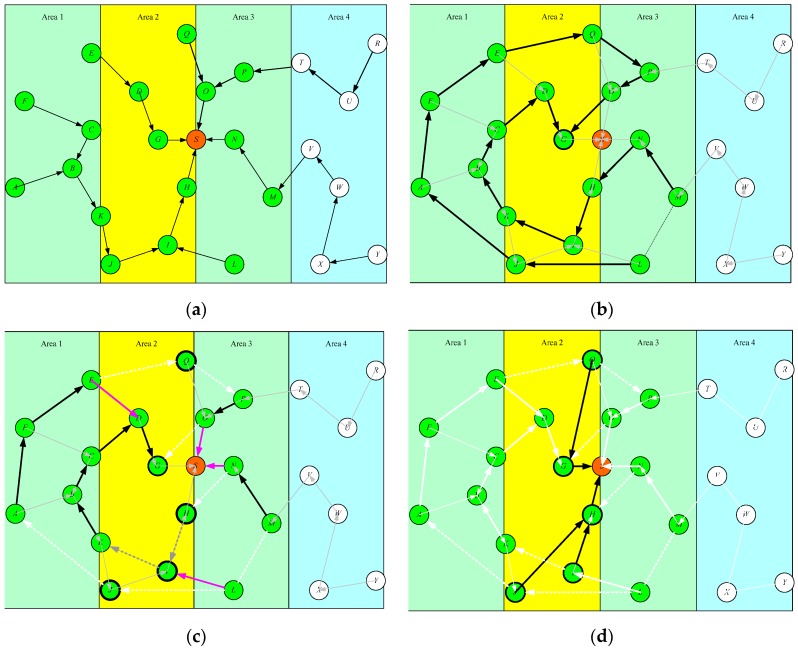
Example of charging area aware multi-mode data gathering method. (**a**) construct the minimum spanning tree of the vertices rooted at the sink; (**b**) type1 and type2 sensors are merged as a chain; (**c**) update the next receivers of the type2 sensors in the constructed chain of (b) to save energy; (**d**) the type1 sensors update their next receivers.

A little example of the CMRP algorithm is shown in Figure 3. There are 24 sensors deployed within the ROI, and the sink is placed at the center of this area shown as Figure 3a. The coverage of sunlight shifts from area1 to area4 periodically. The current sunlight coverage is at area2. Therefore, the sensors located within the area2 are type1 sensors. The sensors located within area 1 and 3 are the type2 sensors, and the type3 sensors are those located within the area 4. Sensors and the sink are treated as the vertices on the plane. The minimum spanning tree of these vertices rooted at the sink is constructed shown as Figure 3a. The type1 and type2 sensors are merged to build the circular chain. The type1 sensor *G* is the first leader of this chain. Figure 3b shows the corresponding data propagating target of each sensor based on the chain-based routing protocol. 

All sensors adjust their propagation target according to Equation (5) to prevent propagating data to the sensors which have low energy replenishing rate. In this example, sensor *N* changes its next receiver from *H* to *S*, sensor *L* changes from *J* to *I*, sensor *E* changes from *Q* to *D*, sensor *J* changes from *A* to *I*, and sensor *O* changes from *G* to *S*. Note that sensor *D* still propagates its data to *G* because both of them are type1 sensors shown as Figure 3c. Finally, the sensor G and H are selected as the CHs from the sensors {*Q*, *G*, *J*, *I*, *H*} which have NULL next receivers. Then, the groups {*G*, *Q*}, and {*H*, *I*, *J*} become two clusters and use the cluster-based routing protocols to propagate data to sink shown as Figure 3d.

## 4. Simulation Results

This section shows the simulation results. A C++ program was implemented for evaluation. The LEACH [5], RICA [24], MSTP [27], and the PEGASIS [11] were also implemented for comparison. The evaluation metrics include the lifetime until the first node die, the lifetime with different percentage of dead nodes, the average number of data propagation hops, the average propagation delay, the average remaining energy until the first node die, and the life-delay rate.

The lifetime until the first node dies and the lifetime with different dead nodes percentage evaluate whether a method can efficiently balance the energy of sensors in the network. The energy consumption on data monitoring is constant. However, data propagation is not because sensors need to take turns to be the CH or leader. The sensors can operate longer if the energy consumption on data propagation can be evenly distributed to all members in the network. The lifetime is computed in the number of rounds and a round is defined as all sensors in the network finish propagating data to sink.

The average number of data propagation hops evaluates whether a method can quickly deliver the data to the target through by using fewer numbers of relay processing. The average data propagation delay indicates the delay time to propagation data to the sink. Note that propagation hops does not imply short propagation delay. If sensors have the same target to receive data, they need to be queued to send data. Therefore, a routing method with fewer numbers of propagation hops does not imply it will have shorter propagation delay. Propagation delay can be reduced if multiple sensors can propagate their data simultaneously.

The average remaining energy until the first node dies indicates whether a routing method can evenly distribute the power consumption on data propagation to all sensors or not. A method makes part of sensors to take the overhead of data propagation will shorten the operation time of the whole network. Therefore, the average remaining energy of sensors is high.

The life-delay rate (LDR) is defined as the product of the number of rounds and the propagation delay. Both values are normalized. Let Rnd(M) be the number of rounds of method M. The normalized number of rounds of a method is defined as Rnd(M)/Rnd(M^*^) where M^*^ is the evaluated method that has the maximum number of operating rounds. The definition of the normalized propagation delay is similar. A method with LDR is close to 1 means it considers both the delay time and operation lifetime.

### 4.1. Environment Setups

Initially, sensors are deployed within a 400 m × 400 m area randomly. The numbers of deployed sensors are {100, 125, 150, 175, 200}. The sink is located at the center of the RoI. The size of the data packet is 2000 bits. A sensor can propagate its data out when it receives all data that needs a route through it to the sink. The initial energy of all sensors is 1 Joule. The energy consumed on data aggregation is set to 5 nJ/bit. The energy to set up the transmitting and receiving circuits is 50 nJ/bit. The energy for the transmitter amplifier to dissipate one data bit is set to 100 pJ/bit/m^2^. The location of sunlight coverage changes periodically. In the simulation of this paper, the sunlight coverage is assumed to be a continuous rectangle area. This area rotates from left side to right side as the daily trajectory of the sun. The default rotating period for moving sunlight coverage to the next area is 150 rounds. The periods with {50, 150, and 600} rounds are simulated to evaluate the impacts of the rotating period. The charging rate of the sensors within the charging area simulated is 0.0001 J per round. In this simulation, 500 random topologies are evaluated to compute the averaged results.

### 4.2. Numerical Results

**Figure 4:**
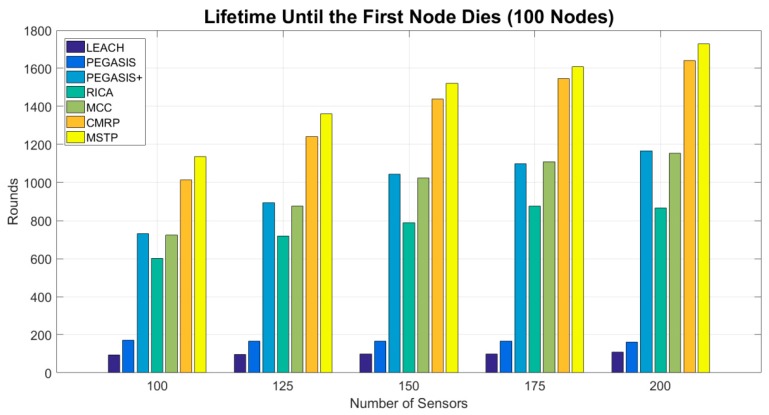
Lifetime until the first node dies.

Figure 4 shows the lifetime until the first node die. The numbers of rounds of the LEACH and PEGASIS methods are lower than 200. The LEACH spends too much energy on long-distance data transmission. The original PEGASIS uses a bad chaining methodology that causes it to introduce worse results. The PEGASIS+ is applying the proposed MCC method to chain the sensors. This chain is created by linking the sensors into a circle at first and then breaks the longest edge. The results become better explicitly. The results of the ring-based method RICA [24] is better than original PEGASIS because it has a better chaining mechanism. When the ring-based method RICA applies the proposed MCC mechanism to chain the sensors into a circle, denoted as MCC, the number of operation rounds increases about 20–30%. A well adjacency methodology explicitly reduces the data transmission distance so that sensors can save their energy and the network has a longer operation time. Among all evaluated methods, the MSTP method has the longest lifetime. MSTP selects the shortest adjacent neighbor to propagate data so that every sensor can minimize the energy consumption used on propagating data to its next hop.

The lifetime of the CMRP is only worse than the MSTP by no more than 1%. It sacrifices some energy to reduce the propagation delay. CMRP considers the lifetime and the propagation delay simultaneously. The sensors with plenty of energy make their effort to reducing the propagation delay, and the sensors located at the unchangeable area shorten their transmission distance to save energy. However, the CMRP exhibits better propagation delay than that of the MSTP in the later experiments.

**Figure 5:**
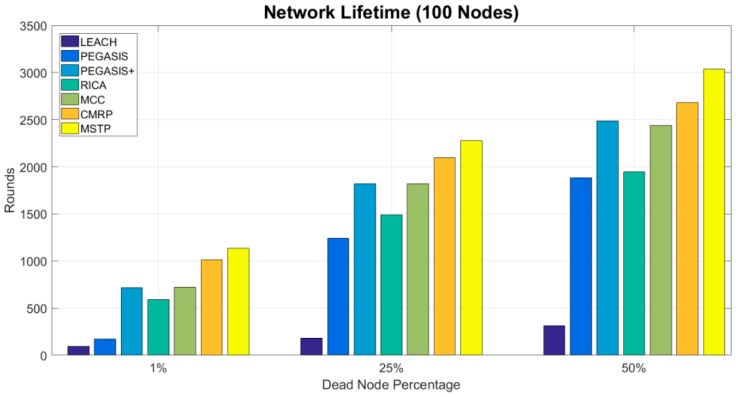
Number of rounds with different dead nodes percentage.

Figure 5 shows the lifetime until 1%, 25%, and 50% of nodes die in a network with 100 sensors. The cases that more than 75% of nodes die are not evaluated because sensors can replenish their energy and part of the method does not have these cases. In the cases of 1% of sensor nodes dying, the numbers of rounds in LEACH and PEGASIS methods are less than 200. However, the PEGASIS method exhibits outstanding results against the LEACH in the cases of 25% and 50% of nodes dying. The original PEGASIS method uses a bad chaining methodology to link the sensors. When those sensors which have long-distance neighbors exhaust their energy, the remaining sensors with shortening distance neighbors can operate longer. Therefore, the number of operation rounds increases explicitly. The RICA uses a better chaining method to link the sensors so that is has better results than the LEACH and PEGASIS. When the MCC mechanism is applied to chain the sensors, the PEGASIS+ and the RICA-like MCC methods address the problem of creating long-distance neighbors for sensors. Therefore, their results are improved explicitly.

The MSTP greedily constructs the closest neighbors for each sensor so that every sensor can prevent wasting their energy on long-distance data propagation. Consequently, it has the best results. The proposed CMRP integrates the advantages of the MSTP, RICA, and cluster-based methods. Although its result is worse than the MSTP, it is still better than the other method. 

**Figure 6:**
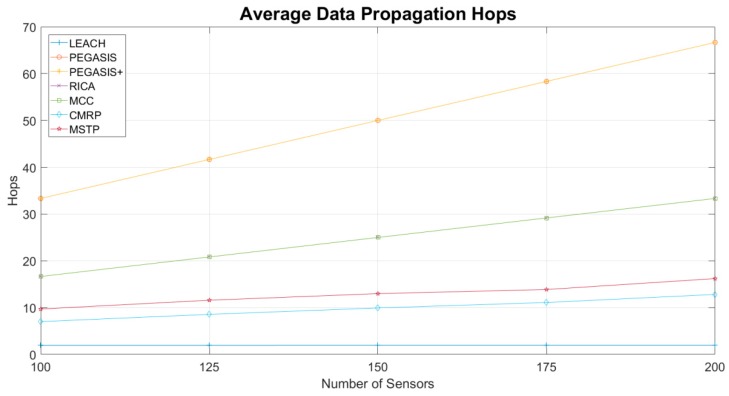
Average number of data propagation hops.

Figure 6 evaluates the average number of propagation hops. The LEACH method delivers the data to sink within two hops. Chaining the sensors into a long chain makes PEGASIS and PEGASIS+ methods introduce a great number of propagation hops. The RICA and the MCC methods reduce the chain of PEGASIS into two small ones with 50% length and make the sensors on these two chains operate simultaneously. The number of propagation hops used by the MSTP method depends on the topology. The average number of propagation hops is within 10–20 and increases as the number of sensors grows. By dynamically changing the data propagation target according to the recharging status of the sensors, CMRP has better results than the MSTP.

**Figure 7:**
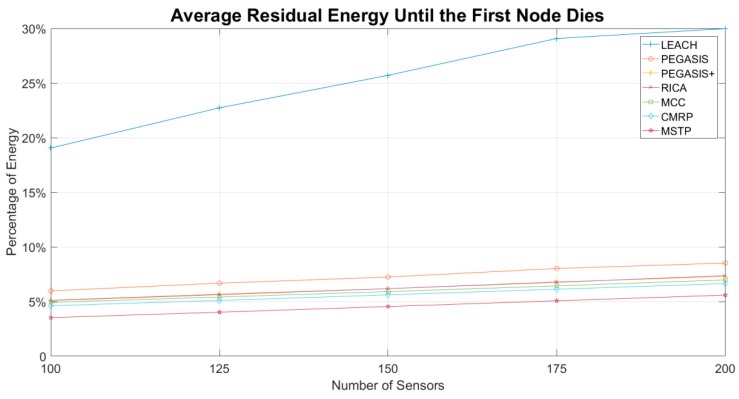
Average residual energy until the first node dies.

Figure 7 shows the average remaining energy of the sensors until the first node dies. Sensors in the LEACH method have fewer propagation hops and have extremely unbalanced energy consumption. The average residual energy of the sensors is more than 18–30%. While the number of sensors increases, the average residual energy of sensors grows. It indicates that sensors do not efficiently share the data propagation overhead.

Except the LEACH method, the average residual energy of sensors in the other methods is within 3–9%. PEGASIS exhibits the worst results because the bad chaining mechanism makes part of sensors select long-distance propagation targets. By applying the proposed MCC chaining mechanism, the average remaining energy of the PEGASIS+ reduces about 1% of the original PEGASIS. It is slightly worse than the RICA. The results of RICA are similar to that of the PEGASIS+. The proposed chaining method makes the results of MCC better than that of RICA. The MSTP exhibits the best results among all evaluated methods. Sensors spend most of their energy on data gathering and propagate data in short distance. These two characteristics mean MSTP has the longest lifetime so that it has the lowest average remaining energy. The average residual energy of CMRP is 1% more than MSTP because the sensors within the sunlight coverage sacrifice part of the energy to reduce the propagation delay.

**Figure 8:**
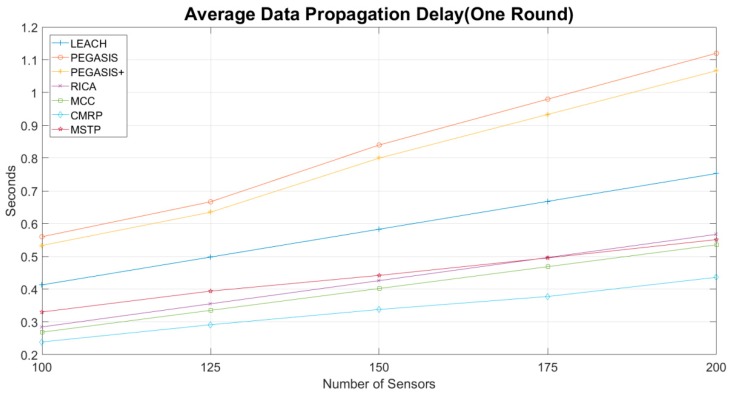
Average data propagation delay.

Figure 8 shows the average propagation delay in a round. In this experiment, the data transmitting rate is assumed to be 1 Mbps and carry sensing is 1 ms. The PEGASIS and PEGASIS+ methods have the worst propagation delay because data need to be delivered one by one on the chain. The propagation delay is explicitly proportional to the number of sensors in the network. At most two sensors can propagate data simultaneously. For the LEACH method, sensors in each cluster need to send their data to the CH one after another. If the network has five clusters, up to five sensors can transfer data simultaneously. The RICA method divides the length of the chain in PEGASIS into two equal size chains. Data in these two chains can be delivered simultaneously so that its data propagation delay is less than the PEGASIS. Similarly, the MCC chains the sensors as the RICA but shortens the distance. Therefore, MCC has less propagation delay than the RICA.

The propagation length of the MSTP heavily depends on the topology. The propagation delay is slightly worse than the RICA when the sensors are less than 175. It is because the children nodes of the same parent in the tree need to send their data in turn. In addition, the MST usually constructs an unbalanced tree. The maximum depth of the tree is usually near 40% number of sensors. Thus, the propagation delay is worse than the RICA. However, densely deployed sensors can reduce the chance to construct an unbalanced MST. The propagation delay is equal or less than the RICA when the number of sensors is more than 175.

The CMRP exhibits the shortest propagation delay. In the CMRP method, about 25–50% sensors use the spanning tree method, 25–50% use the ring-like method to propagate data, and 25% sensors use the cluster-based method to delivering the data. CMRP still has the lowest delay time even the number of sensors increases. The propagation delay of CMRP is less than MSTP about 21–28%.

**Figure 9:**
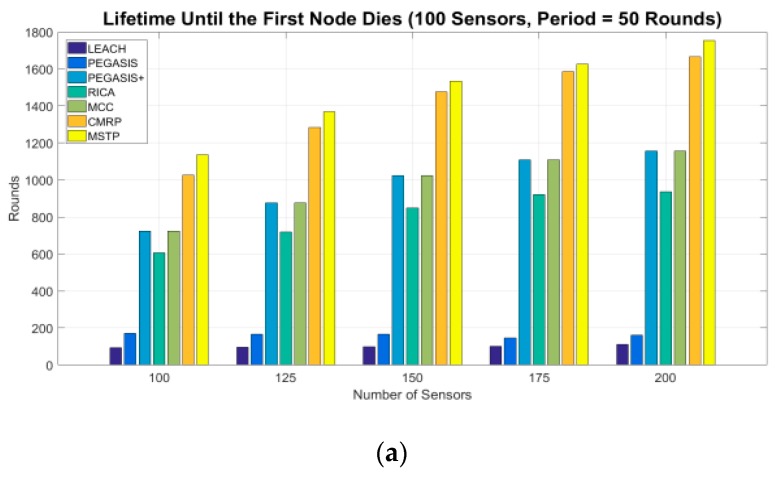

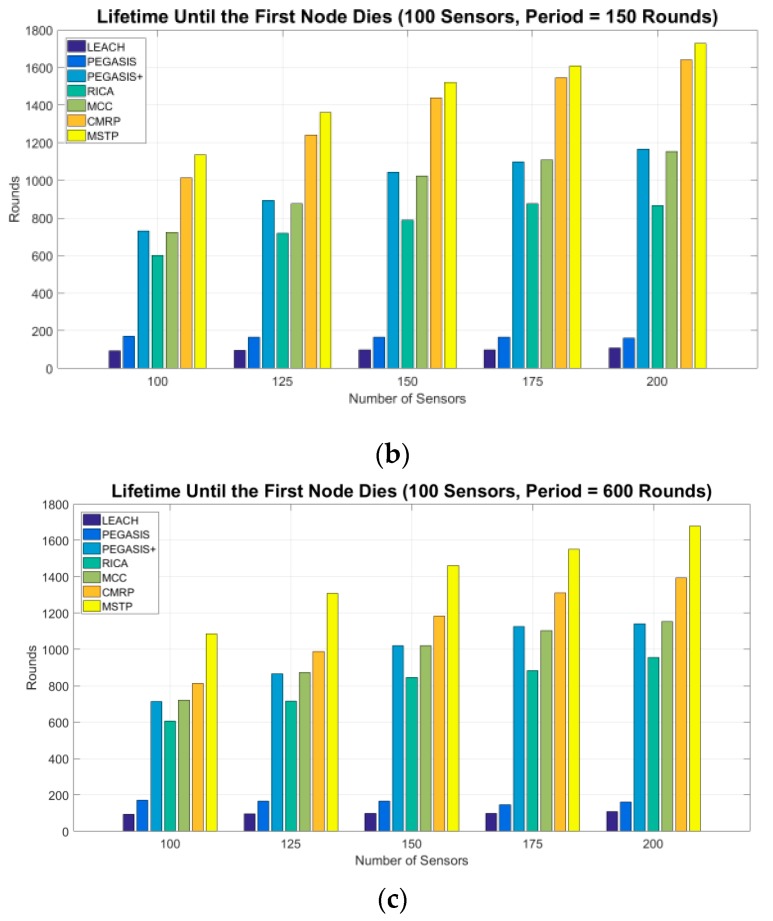
Impact of the sunlight coverage period. (**a**) coverage period is 50 rounds; (**b**) coverage period is 150 rounds; (**c**) coverage period is 600 rounds.

**Figure 10:**
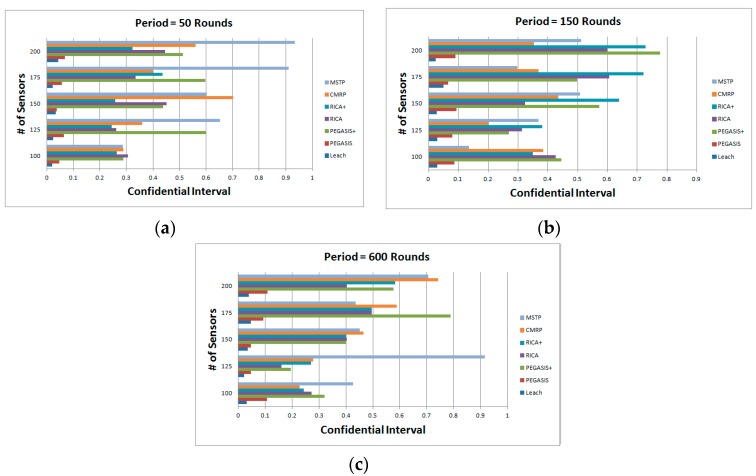
Confidential interval with alpha = 0.95 of Figure 9. (**a**) confidential interval of Figure 9a; (**b**) confidential interval of Figure 9b; (**c**) confidential interval of Figure 9c.

Figure 9 shows the impact of the sunlight coverage period. Three period {600, 150, 50} rounds are simulated shown in Figure 9a–c, respectively. When the period shortens, the numbers of operation rounds of all methods slightly increases except the CMRP. The improving rate of the operation rounds is no more than 5%. However, the improving rate of the CMRP is ranged from 15–29%. The CMRP adapts the routing mechanism according to the charging status of the sensors. The intelligent adapting makes it suitable for application in rechargeable wireless sensor networks. The results of the confidential interval with alpha = 0.95 of Figure 9a–c are shown in Figure 10a–c. Most of the results have no more than 0.8% error.

Finally, the life-delay rate is listed in Table 1. The LEACH does not consider the energy and the delay time at the same time so that the LDR is far away to 1. Without effective scheduling of the adjacent sensors, the original PEGASIS exhibits worse LDR value as the LEACH. However, the LDR of the PEGASIS+ is 9-times that of the original PEGASIS. The RICA is designed to reduce the propagation delay of the PEGASIS and is tried to make two groups of sensors transfer data at the same time. Therefore, its LDR value approximately 1.5-times that of the PEGASIS+.

The MSTP devotes its efforts on reducing energy consumption on data propagation. It makes multiple sensors to transfer data at the same time so that the LDR is higher than 0.7. The CMRP takes advantage of the MSTP to reduce the energy consumption for extending the operation time of the energy-lacking sensors. Sensors with moderate energy resource use the ring-based method to reduce the energy consumption and propagation delay. Sensors with plenty of energy resource use the cluster-based method to reduce the propagation delay. Thus, the CMRP has the best value on LDR.

## 5. Conclusions

A charging-aware multi-mode routing protocol based on the charging status of sensors is proposed for data gathering. CMRP is designed for the scenario that sensors can replenish their energy. Sensors with plenty of energy select the routing methods which introduce shorter propagation delay time and the sensors with less energy apply the routing method that consumes less energy. The next propagation target of CMRP considers both the charging status of the sensors and the distance to the sink. Simulation results show that the proposed CMRP can reduce energy consumption and shorten the propagation delay at the same time. The life-delay rate of the CMRP is better than all the other evaluated method. In future work, the gradually changed sunlight coverage can be used so that the CMRP can be applied to the scenario that sensors have different charging rate.

## Figures and Tables

**Table 1:** LDR value of the evaluated methods.

	Number of Nodes	100	125	150	175	200
Protocols						
LEACH		0.047318	0.041506164	0.037179	0.034871	0.036515
PEGASIS		0.064274	0.053254316	0.044155	0.039632	0.036429
PEGASIS+		0.284873	0.294922576	0.289819	0.28729	0.272922
RICA		0.43765	0.416311686	0.426872	0.414078	0.384849
MCC		0.565505	0.558406847	0.565445	0.555464	0.543804
CMRP		0.892515	0.911732311	0.946629	0.961023	0.949263
MSTP		0.723171	0.739505321	0.764615	0.762257	0.791184
